# Abdominal Compartment Syndrome—When Is Surgical Decompression Needed?

**DOI:** 10.3390/diagnostics11122294

**Published:** 2021-12-07

**Authors:** Dan Nicolae Păduraru, Octavian Andronic, Florentina Mușat, Alexandra Bolocan, Mihai Cristian Dumitrașcu, Daniel Ion

**Affiliations:** 1 Carol Davila University of Medicine and Pharmacy, General Surgery Department, University Emergency Hospital of Bucharest, 050098 Bucharest, Romania; dan.paduraru.nicolae@gmail.com (D.N.P.); octavian.andronic@umfcd.ro (O.A.); bolocan.alexa@gmail.com (A.B.); dr.daniel.ion@gmail.com (D.I.); 2 Carol Davila University of Medicine and Pharmacy, Obstetrics and Gynecology Department, University Emergency Hospital of Bucharest, 050098 Bucharest, Romania; mihai.dumitrascu@umfcd.ro

**Keywords:** abdominal compartment syndrome, intra-abdominal pressure, decompression laparotomy, acute pancreatitis, abdominal aortic aneurysm, severe burn

## Abstract

Compartment syndrome occurs when increased pressure inside a closed anatomical space compromises tissue perfusion. The sudden increase in pressure inside these spaces requires rapid decompression by means of surgical intervention. In the case of abdominal compartment syndrome (ACS), surgical decompression consists of a laparostomy. The aim of this review is to identify the landmarks and indications for the appropriate moment to perform decompression laparotomy in patients with ACS based on available published data. A targeted literature review was conducted on indications for decompression laparotomy in ACS. The search was focused on three conditions characterized by a high ACS prevalence, namely acute pancreatitis, ruptured abdominal aortic aneurysm and severe burns. There is still a debate around the clinical characteristics which require surgical intervention in ACS. According to the limited data published from observational studies, laparotomy is usually performed when intra-abdominal pressure reaches values ranging from 25 to 36 mmHg on average in the case of acute pancreatitis. In cases of a ruptured abdominal aortic aneurysm, there is a higher urgency to perform decompression laparotomy for ACS due to the possibility of continuous hemorrhage. The most conflicting recommendations on whether surgical treatment should be delayed in favor of other non-surgical interventions come from studies involving patients with severe burns. The results of the review must be interpreted in the context of the limited available robust data from observational studies and clinical trials.

## 1. Introduction

Compartment syndrome occurs when increased pressure inside a closed anatomical space compromises tissue perfusion [1]. In the human body there are multiple inextensible anatomical compartments in which the increase in pressure causes changes in homeostasis by directly or indirectly decreasing the vascular supply to the tissues [1]. Examples of anatomical spaces are the cranial box, the orbit, the thoracic cavity, the pericardium, the abdominal cavity, and the musculoskeletal compartments of the upper and lower limbs. The sudden increase in pressure inside these spaces requires rapid decompression by means of surgical intervention.

Compartment syndrome was first described in the lower limbs in 1811 by German surgeon Richard von Volkman in Centralblatt für Chirurgie [2]. The characteristics of abdominal compartment syndrome (ACS) were first described in 1984 by I. Kron, P.K. Harman and S.P. Nolan [3]. However, the terminology of “abdominal compartment syndrome” was introduced only five years later, by Fietsam et al. [4]. Throughout this time span of about two centuries, numerous studies have been conducted on methods of measuring pressure in the abdomen, its influence on respiratory [5] and cardiovascular [6] systems, and the effects of closing the abdomen in tension [7,8]. In 2006, following the International Conference of Experts on Intra-Abdominal Hypertension and Abdominal Compartment Syndrome [9], the definitions of the concepts of intra-abdominal hypertension (IAH) and ACS were established, and one year later a series of recommendations were formulated regarding the management of these entities. These guidelines were last updated in 2013 [10] and at present ACS is defined as a sustained intra-abdominal pressure (IAP) >20 mmHg (with or without an abdominal perfusion pressure (APP) <60 mmHg) that is associated with new organ failure.

In the case of abdominal compartment syndrome, surgical decompression consists of a laparostomy that can be performed using several techniques, most often by median laparotomy extended from the pubis to the xiphoidal process [11,12]. Another method of laparotomy consists of an extended transverse incision in the flanks placed a few centimeters below the costal margin. The third option of surgical decompression involves making 3 transverse incisions 2–3 cm long located on the midline through which the white line will be sectioned vertically leaving the peritoneum intact [13]. Regarding decompression laparotomy (DL), the indications from World Society of Abdominal Compartment Syndrome (WSACS) are limited. To summarize, decompression laparotomy is recommended in cases of overt ACS in critically ill adults with ACS. In the diagnosis of IAH/ACS, establishing the indication of DL and the appropriate time for performing it is an important step. Early surgery can have an overall unfavorable impact due to surgical stress, while delaying this procedure can produce irreversible complications with fatal potential. The criteria for defining IAH/ACS are relatively clear and intensively studied, while for establishing the surgical indication as part of diagnostic management there are no standardized protocols or algorithms.

The aim of this review is to identify the diagnostic landmarks and indications for the appropriate moment to perform decompression laparotomy in patients with ACS based on available published data.

## 2. Materials and Methods

A targeted literature review was conducted on indications for DL in ACS. The search was focused on three conditions characterized by a high ACS prevalence, namely acute pancreatitis, ruptured abdominal aortic aneurysm and severe burns. Articles published in the last 15 years which reported the use of DL in the context of ACS were included. Case reports, articles not written in English and conference abstracts were excluded. The research was conducted on PubMed and Web of Science databases using the following keywords: abdominal compartment syndrome, intra-abdominal pressure, intra-abdominal hypertension, DL, acute pancreatitis, abdominal aortic aneurysm, and severe burn.

## 3. Results and Discussion

There is still a debate around the clinical characteristics which prompt for surgical intervention in ACS. WSACS guidelines include a recommendation for attempting to lower the IAP by means less invasive than a laparotomy, such as percutaneous catheter drainage. A study comparing percutaneous catheter decompression with open abdominal decompression on 62 patients divided equally according to the two types of treatment showed that failure to drain at least 1000 mL of fluid and decrease the patient’s IAP by at least 9 mm Hg within the first 4 h following PCD should prompt early surgical decompression to improve the patient’s survival chances from IAH/ACS. Selected patients developed ACS in the context of general, vascular, or oncological surgery (36%), trauma (23%), sepsis or multiple organ failure (12%) and burns (29%) [14].

ACS was classified by the WSACS based on the underlying mechanism into primary ACS or ACS secondary to a pre-existing condition [10,15]. Primary ACS is a complication of injuries located in the abdomino-pelvic region, whereas secondary ACS occurs in the context of conditions that affect other regions of the body. In general, in patients with abdominal trauma, ACS is the consequence of clinical situations which can sometimes coexist, such as massive volume resuscitation with consecutive visceral edema, the presence of retroperitoneal hematoma, hemostatic packing performed during damage control laparotomy (DCL), post-injury bowel paresis, and associated third degree burn of the abdominal wall [16,17,18]. In patients with abdominal trauma, the indication for laparotomy is given primarily by life-threatening visceral injuries regardless of intra-abdominal pressure.

Also, in order to combat the lethal triad represented by acidosis (pH ≤ 7.2, lactate levels ≥ 5 mmol/L, base deficit (BD) ≥ −6), hypothermia (≤34 °C) and coagulopathy (blood loss ≥ 4 L during the operation, and/or transfusion requirement ≥ 10 U of packed red blood cells, INR/PT > 1.5 times normal) surgeons introduced into practice the concept of DCL [19]. DCL practice involves three stages, starting with laparotomy to control bleeding and sources of intra-abdominal contamination completed by methods of temporary closure of the abdomen, continuing with a period of resuscitation and rebalancing of the patient in the intensive care unit and ending with surgery and permanent parietoraphy [11,20]. Thus, the second stage of DCL, namely the open abdomen period, represents a strategy to prevent ACS in trauma patients, but does not represent the topic of the current research.

We will further discuss the indications for DL in two examples of primary ACS (acute pancreatitis and ruptured abdominal aortic aneurysm) and in one example of secondary ACS (extensive burn lesions).

### 3.1. Decompression Laparotomy in ACS from Acute Pancreatitis

ACS is one of the complications of severe acute pancreatitis, with an incidence of 4–27% [21,22,23]. However, the timing, indications and threshold value for surgical decompression are controversial and current evidence is unclear in terms of which approach should be selected in any particular setting (Table 1).

A meta-analysis [30] that includes seven studies performed between 2003 and 2012 on 103 patients with acute pancreatitis complicated by ACS reports surgical decompression in 76 cases (73%) either as first intervention or after percutaneous catheter drainage of intra-abdominal fluid. Of the 11 patients who initially underwent PCD, 8 patients subsequently required DL. Surgical decompression consisted in most cases of a median laparotomy (*n* = 66), but subcutaneous white line fasciotomy (*n* = 17), or full thickness transverse bilateral subcostal laparotomy (*n* = 1) were also performed. A decrease in IAP was reported in 60 cases, from a median initial IAP value of 33 mmHg to 18 mmHg.

The only randomized study [31] (DECOMPRESS TRIAL) that aimed to determine whether surgical decompression should be the first-line therapeutic measure for patients with ACS in the context of severe acute pancreatitis currently has no results available. The study’s authors hypothesized that DL with temporary abdominal closure will decrease overall mortality and major morbidity in patients with abdominal compartment syndrome during acute pancreatitis compared with percutaneous puncture and placement of the abdominal catheter.

Another retrospective study [29] comparing 212 patients who underwent percutaneous catheter drainage (PCD) with a group of 61 patients who underwent open laparotomy with temporary closure for the treatment of ACS in the context of severe acute pancreatitis indicates that PCD is associated with superior results compared to surgical decompression in terms of complications and mortality rate. This study also states that the indications for PCD conversion to DL were: absence of clinical improvement of IAP three days after decompression, lack of experience in correct PCD catheter positioning, massive intra-abdominal hemorrhage and intestinal perforation requiring emergency laparotomy.

Based on a retrospective study, Chen et al. [23] consider that decompression by invasive methods in acute pancreatitis should be considered starting from IAP values of 20–25 mmHg, without waiting to reach values of 30–40 mmHg. A delay in establishing invasive decompression procedures from the moment of ACS installation could potentially lead to bacterial invasion of the pancreas through the intact intestine, due to splanchnic ischemia-reperfusion syndrome. This argument is supported by significantly higher rates of pancreatic infection, septic shock, multiple organ dysfunction syndrome and mortality in the group of patients with acute pancreatitis who developed ACS, compared to the group of patients who did not develop this complication. In this study, invasive decompression was established at the mean IAP value of 36.69 ± 5.33 mmHg and at an average interval of 28.38 ± 2.29 h from the occurrence of ACS. The authors also recommend rebalancing hypovolemia, acidosis, and coagulation disorders before any invasive decompression intervention.

### 3.2. Decompression Laparotomy in ACS after Ruptured Abdominal Aortic Aneurysms (AAA) Repair

There are 2 techniques for repairing a ruptured abdominal aortic aneurysm (AAA): the open technique and the endovascular technique. Both of them are associated with the risk of postoperative ACS, either due to the high amount of fluids administered in open repair or to the retroperitoneal hematoma from the endovascular repair. The incidence of ACS after endovascular aneurysm repair for ruptured abdominal aortic aneurysms varies significantly in the literature of the last 20 years. On average, the incidence is about 9%, ranging between 0% and 40% [32] (Table 2).

In a retrospective study which included 12 patients with AAA for whom EVAR was performed, Ko et al. [33] identified three patients who developed ACS in the first 48 h after the procedure. The diagnosis of ACS and the indication for DL were not established on the basis of PIA measurements, but on clinical-paraclinical arguments, such as decrease in blood pressure, decrease in hemoglobin value and organ dysfunction. Moreover, the authors report a high level of suspicion for ongoing bleeding, which dictated the decision for immediate laparotomy. Both of the patients who underwent DL at an early stage of ACS survived. The only mortality case was represented by the patient with ACS who refused surgical intervention.

The largest study on ACS after AAA repair included 8765 patients with ruptured AAA, 120 of whom developed ACS postoperatively [34]. This prospective study shows that the high mortality of patients with ACS is not influenced by the timing of DL or by the main pathophysiological findings such as post-operative bleeding, bowel ischemia, or edema. Also, there were no statistically significant differences between survivors and non-survivors regarding duration of IAP >15 or >20 mmHg before DL, maximum IAP and IAP after DL.

Miranda et al. [35] identified three risk factors that are associated with ACS development after ruptured AAA in patients who have undergone EVAR, namely preoperative and intraoperative transfusion of 3 or more units of packed red blood cells, a postoperative hemoglobin <8 g/dL, and a systolic blood pressure <86 mm Hg on presentation.

Decompressive laparotomy for ACS can also be performed during endovascular repair of ruptured abdominal aortic aneurysms, but Adkar et al. [36] concluded that it is associated with a significantly worse 30-day survival rate.

Rubenstein et al. [39] suggest that early development of ACS after EVAR is a sign of continued hemorrhage from lumbar and inferior mesenteric vessels through the ruptured aneurysm sac. In this event, immediate DL and ligation of the vessels are necessary.

In an attempt to identify a marker to diagnose the onset of ACS early, Horer et al. [42] proposed to calculate the lactate/pyruvate ratio and the value of glycerol in the peritoneal fluid obtained from the peritoneal microdialysis technique. In his study, Horer compared a group of patients who underwent DL with a group of non-decompressed patients in terms of lactate/pyruvate levels and glycerol levels after REVAR. In the group of decompressed patients, one patient had IAH grade I, one had IAH grade II, 3 had IAH grade III and one had IAH grade IV. In this study, the authors demonstrate the existence of metabolic changes in the peritoneal fluid that precedes the installation of ACS and that could be used as an indication for performing DL. In contrast to the early DL practiced in the study of Horer et al., Djavani Gidlund et al. [43] suggests that after AAA repair, IAP should be monitored every 4 h and medical treatment should be initiated immediately if the IAP exceeds 12 mmHg. In that study, patients with IAP >12 mmHg post EVAR were managed by a series of conservative measures following which only six of 16 patients exceeded the IAP value of 20 mmHg and only three developed ACS.

### 3.3. Decompression Laparotomy in ACS from Extensive Burn Lesion

Among the complications that develop in patients with burns >15% total body surface area (TBSA), ACS occurs with a prevalence of 4.1–16.6%, and is most often associated with burns on >70% TBSA [47]. There are no clinical trials to indicate the optimal treatment for patients with ACS secondary to severe burns, and studies to date include a small number of patients [48,49,50,51,52,53,54] (Table 3). The groups of patients who underwent DL usually include both pediatric and adult patients, most with burns >50% TBSA and a mean IAP pre-decompression >40, in whom conservative treatment instituted for at least 24 h failed (Table 3). The post-laparotomy survival of decompression reported by the studies identified so far varies between 0% and 66% [48,49,50,51,52,53,54].

The first report of a DL for the treatment of secondary ACS in patients with burns was in 2002 by Hobson et al. [48], with a survival rate of 48% after this procedure. Later studies (2002 to 2009) reported a very low survival rate in this population undergoing DL ranging from 0% to 16% [49,50,51].

Based on a retrospective study performed on 25 patients with burns (mean 64.6 ± 3.9% TBSA) who developed ACS and required emergency laparotomy in the first 24 h, Hershberger et al. [50] proposed an ACS management algorithm secondary to burns. The most important risk factor in the occurrence of ACS in burned patients is hydro-electrolytic resuscitation by infusion solutions. Thus, Hershberger et al. states that when the patient has been hydrated with a total of over 200 mL/kg body, certain measures must be instituted to prevent ACS. These include performing escharotomies or supplementing existing ones, reducing intravenous hydration and administering albumin. If after these measures the IAP exceeds 30 mmHg and the diuresis per hour does not improve, a percutaneous drainage catheter should be placed in the peritoneal cavity. Only when the patient’s clinical condition does not improve after all these steps is it recommended to perform DL. In this study, the mean age of the patients was 28 ± 3.8 years (seven patients were under 18 years of age), the mean intra-abdominal pressure before decompression was 57 ± 4.2, the mean time from burning to the time of DL was 13.3 ± 1.3 h, and mortality was 88% (22 of 25 patients).

The largest study group was published by Ramirez et al. [52] in 2018 and included 41 patients. This study documents the highest reported value for the survival rate of 66%. According to this study, the most important factors influencing the mortality of patients with ACS after DL are the timing of laparotomy, the timing of subsequent operations, and the timing of abdominal closure. Ramirez et al. argue that DL should be performed early, within the first hour after the diagnosis of ACS. Moreover, the authors claim that delaying the surgery in order to institute conservative treatment of ACS (assuring a patent bladder catheter, insertion of a nasogastric tube, escharotomies of any existing circumferential abdominal wounds, sedation, pharmacological paralysis [56]), as practiced in previous studies have resulted in increased mortality.

On the other hand, Oda claims from a study conducted in 2007 on 38 patients that DL has an unfavorable impact on the evolution of burned patients, aggravating multiple organ failure and acute long-term injury [55]. Some authors consider that DL should even be avoided in certain categories of patients, such as those over 80 years of age who have a higher mortality rate than young people [57].

## 4. Conclusions

The results of our research aimed at introducing the indications for DL in the diagnostic protocols of IAH/ACS show that DL is an effective intervention for reducing IAP and has an immediate beneficial effect on organ function. This procedure should be considered in patients with ACS and remains one of the most important means to address both primary and secondary ACS. However, indications and timing for DL are highly dependent on the particularities of the underlying pathology. The only consensus available at this moment is on performing DL when there is no improvement in the clinical status of the patients after attempting several conservative measurements. It is still unclear as to how much time a clinician should wait from the onset of the ACS to the initiation of the conservative treatment and what is the IAP threshold value beyond which surgical intervention is necessary. There is also the question of how much time the conservative treatment should be applied before advancing to more invasive procedures.

According to the limited data published from observational studies, laparotomy is usually performed when IAP reaches, on average, values ranging from 25 to 36 mmHg in the case of acute pancreatitis. In cases of a ruptured abdominal aortic aneurysm, there is a higher urgency to perform DL for ACS due to the possibility of continuous hemorrhage. The most conflicting recommendations on whether surgical treatment should be delayed in favor of other non-surgical interventions come from studies involving patients with severe burns.

The results of the review must be interpreted in the context of the limited available robust data from observational studies and clinical trials.

## Figures and Tables

**Table 1:** Abdominal compartment syndrome in the context of acute pancreatitis in different studies. ACS—abdominal compartment syndrome; DL—decompression laparotomy; IAP—intra-abdominal pressure; IAP1—intra-abdominal pressure before decompression; IAP2—intra-abdominal pressure after decompression; Δ IAP—decrease in IAP after decompression; nr—not reported; SLAF—subcutaneous linea alba fasciotomy.

	Study Type	Pancreatitis (N)	ACS (*n*)	DL (*n*)	IAP_1_ (mmHg)	IAP_2_ (mmHg)	Δ IAP	Timing to DL	Decompression Technique
De Waele JJ, 2005 [24]	Prospective	44	4	4	>25	nr	19	nr	Midline Laparotomy
Leppäniemi, 2011 [25]	Retrospective	10	10	10	31 (23–45)	20 (10–33)	10	nr	Subcutaneous linea alba fasciotomy
Mentula, 2010 [26]	Retrospective	26	26	26	31.5 (27–35)	nr	16 (9–21) after midline laparotomy	>5 days (9 cases) from pancreatitis onset—no survivors 1–4 days (17 cases) from pancreatitis onset—14 survivors	midline laparotomy—18 patients, transverse bilateral subcostal laparotomy—1 patient SLAF—7 patients, 2 of whom underwent completion midline laparotomy on postoperative day 1
Bezmarevic, 2012 [27]	Prospective	51	6	5	21.2 (20–23)	nr	nr	1–4 days	Midline Laparotomy
Chen, 2008 [23]	Retrospective	74	20	5	36.69 ± 5.33	18.31 ± 3.25	18	28.38 ± 2.29 h	Midline Laparotomy
Davis, 2013 [28]	Retrospective	45	16	16	29.5	nr	nr	3.1 h	Midline Laparotomy
Peng T, 2016 [29]	Retrospective, comparative	61	61	61	nr	nr	15	63 h (range, 2–101 h)—from pancreatitis onset	Midline Laparotomy

**Table 2:** Abdominal compartment syndrome in the context of ruptured aortic aneurysm. AAA—aortic abdominal aneurysm; ACS—abdominal compartment syndrome; DL—decompression laparotomy; nr—not reported; REVAR—endovascular aneurysm repair.

	Study Design	Patients with Ruptured AAA (*n*)	Patients with ACS (*n*)	DL (*n*)	Laparotomy Timing	Mortality of Patients with ACS
Ko, 2019 [33]	retrospective	12	3	2	first 48 h after the procedure	33%
Ersryd, 2019 [34]	prospective	8765	120	117	<24 h after AAA repair in 56 (48.7%) 24–48 h in 30 (26.1%) >48 h in 29 patients (25.2%)	50%
Miranda, 2018 [35]	retrospective	25	3	3	immediately	67%
Adkar, 2017 [36]	retrospective	1241	91	91	during REVAR	60%
Papazoglou, 2017 [37]	retrospective	2	3	1	immediately	66%
Oyague, 2015 [38]	retrospective	25	6	nr	nr	100%
Rubenstein, 2015 [39]	retrospective	73	21	nr	nr	62%
Fossaceca, 2014 [40]	retrospective	44	5	5	nr	0%
Mehta, 2013 [41]	retrospective	136	17	nr	nr	59%
Horer, 2013 [42]	prospective	15	6	6	12 h (5–33 h)	16%
Djavani Gidlund, 2011 [43]	prospective	29	3	2	>12 h	33%
Hsiao, 2011 [44]	retrospective	6	1	1	4 days after AAA	0%
Saqib, 2012 [45]	prospective	148	15	15	nr	nr
Noorani, 2012 [46]	prospective	102	1	1	nr	nr

**Table 3:** Abdominal compartment syndrome in the context of burns. ACS—abdominal compartment syndrome; IAP1—intra-abdominal pressure before decompression laparotomy; IAP2—intra-abdominal pressure after decompression; nr—not reported; SLAF—subcutaneous linea alba fasciotomy; TBSA—total body surface area, DL—decompression laparotomy.

	Study Type	Total No.	Pediactric	Adults	%TBSA Burned	IAP_1_	IAP_2_	Conservative Treatment Attempted	DL Timing	Mortality
Hobson, 2002 [48]	retrospective	8	4	4	71%	40 ± 10	26 ± 5	yes	>24 h	62%
Latenser, 2002 [49]	retrospective	4	0	4	>80%	34 ± 6	30 *	yes	>24 h	100%
Hershberger, 2007 [50]	retrospective	25	7	18	64.6 ± 3.9%	57 ± 4.2	nr	yes	13.3 ± 1.3 h	88%
Oda, 2007 [55]	retrospective	14	nr	nr	78.5 ± 10.6%	47 ± 11	20 ± 10	yes	nr	nr
Markell, 2009 [51]	retrospective	32	nr	nr	nr	nr	nr	nr	nr	84%
Ramirez, 2018 [52]	retrospective	41	31	15	62% (children) 58% (adults)	28 (children) 43 (adults)	nr	nr	1 h	44%
Boehm, 2019 [53]	retrospective	38	nr	nr	50%	nr	nr	nr	>24 h	84%
Wise, 2016 [54]	retrospective	3	nr	nr	39.6 ± 26.4%	nr	nr	yes	>24 h	66%

* reported in only 1 patient.

## Data Availability

Not applicable.

## Abbreviations

ACS	abdominal compartment syndrome
IAH	intra-abdominal hypertension
APP	abdominal perfusion pressure
BD	base deficit
DL	decompression laparotomy
DCL	damage control laparotomy
WSACS	World Society of Abdominal Compartment Syndrome
IAP	intra-abdominal pressure
SLAF	subcutaneous linea alba fasciotomy
PCD	percutaneous catheter drainage
AAA	aortic abdominal aneurysm
REVAR	endovascular aneurysm repair
TBSA	total body surface area
